# Pot-Pourri

**Published:** 1894-06

**Authors:** Thomas J. Pugh

**Affiliations:** Hearne, Texas


### Correspondence.

### POT-POURRI.

HEARNE, TEXAS, May 5, 1894.

*Editor Texas Medical Journal:*

In the incipient stage of pneumonia, when the breathing is labored, the pulse rapid, skin dry, temperature high, I have found one-tenth grain pilocarpine, combined with one-fourth grain morphia, to be sufficient to bring about resolution, and put the patient on the road to speedy recovery. Of course the alimentary tract must be looked after, and indications filled, and there is nothing better for this purpose than six grains calomel in two grain doses, one dose every hour and a half until all are taken, followed, if necessary, by castor oil and turpentine. This is my abortive treatment for pneumonia.

In profound cases of hysteria, wherein the patient becomes cold, pulse slow, and complains of internal pains radiating from the uterus,—generally to the left side,—with ice-cold extremities, simulating congestive chill, I have found nothing in the range of materia medica that will bring such a case around more quickly than one-tenth grain apomorphia, hypodermically administered. Emesis is produced in two minutes, the patient is relaxed, her hysterical convulsions and rigidity of body are gone. For after, and preventive treatment, I would put the patient on penta-bromides, Peacock's bromides, and celerina, combined in equal parts.

Just here I want to claim priority in the use of apomorphia in asphyxia by inhalation of gas. A case in point: Last June, while attending the National Railway Surgeons' Convention, at Omaha, Nebraska, one morning, just after breakfast, Drs. Dewey and Talley, of Missouri, and myself were sent hurriedly up the elevator of the Barker hotel to see a man who at 11 o'clock the previous night had blown out the gas in his room, and as a result, at 8 o'clock the next morning was found pulseless, breathless, and apparently lifeless. After all except myself had suggested a whiskey drench, which failed on trial, I asked Dr. Dewey if he had his hypodermic syringe and a vial of apomorphia pellets. Responding in the affirmative, he kindly handed me his instrument—a Parke-Davis aluminum syringe, which, by the way is one of the best on the market. I administered hypodermatically two one-tenth grain pellets of apomorphia. Within three minutes, the man was vomiting copiously, his respiration and circulation restored. In half an hour he was pronounced out of danger, and two hours afterwards left the hotel,—a wiser man, no doubt, than when he went there.

Whilst in this field, thinking of the great usefulness of this drug, I would say I would not hesitate to use it in a case of drowning, as I am quite sure it would produce all the results that could be produced by artificial respiration, and possibly more. If reflex action be not destroyed, life would certainly rally its force through the instrumentality of this great drug, which is simply the emetic principle of opium.

THOMAS J. PUGH, M. D.

